# 

**Published:** 1864-02

**Authors:** 


### CONTENTS.

### ORIGINAL CONTRIBUTIONS.

ART. VI.—Camp Typhoid Fever. By Wm. S. Edgar, Surgeon 32d Regt. Ill. Vols., ........ 65
ART. VII.—Bright's Disease of the Kidney. By John Bartlett, M.D., of Chicago, (Read before the Chicago Medical Society,)........ 71
ART. VIII.—Nitrous Oxide, or "Laughing Gas," as an Anæsthetic. By E. Andrews, M.D., Prof. of Surgery in Chicago Medical College, 78

### SELECTIONS.

Notice of the Spotted Fever, as it occurred at Newport, Rhode Island, in the Months of January, February, March, and April, 1863, with a History of the Disease, its Symptoms, Diagnosis, and Treatment, By Philip S. Wales, M.D., Surgeon U.S.N., ........ 81
On the Brain of a Bushwoman. By John Marshall, F.R.S., Surgeon to University College Hospital, ........ 100
Disease termed "Black Leg," among the Ottawa Lumbermen,........ 102
Internal Administration of Belladonna in Cases of Severe Burn, ........ 104
Sarracenia Purpurea In Variola. By Noah C. Levings, M.D., of New York, ........ 105
Drought in the West Indes,........ 106

### EDITORIAL.

Illinois State Medical Society, ........ 107
Chicago Medical College Commencement,........ 107
Books and Pamphlets,........ 107
Sarracenia Purpurea in Small-Pox, ........ 107
Commencement in Rush Medical College, ........ 107
Statistics of Mortality in Chicago,........ 108
Dr. Hoffman, of London, and the Chair of Chemistry at Berlin and Bonn, 110
State Board of Examiners for the Degree of Doctor in Medicine,........ 111
Presidency of Guy's Hospital, ........ 118
Death of Leonidas M. Lawson, M.D., ........ 119
French and English Army Hospitals,........ 121
Azulene of Patchouly,........ 122
On Topicial Injections of Strychnine in Cases of Paralysis of the Facial Nerve, ........ 123
New American Pharmacopœia,........ 123
Gossip—New Mode of Preparing Beef Tea,........ 123
Health in the British Army, ........ 124
Scanzoni on Chronic Metritis, ........ 124
Proportion of Births to Population,........ 124
Alleged Poisoning by Strychnine, ........ 125
New Vaccination Act, ........ 125
Two New Cases of Syphilis Conveyed by Vaccination, ........ 125
The Queen and the Tobacco Question, ........ 125

